# Asteroid surface impact sampling: dependence of the cavity morphology and collected mass on projectile shape

**DOI:** 10.1038/s41598-017-10681-8

**Published:** 2017-08-30

**Authors:** Bin Cheng, Yang Yu, Hexi Baoyin

**Affiliations:** 1 Tsinghua University, Beijing, 100084 China; 2 Beihang University, Beijing, 100191 China

## Abstract

*In-situ* exploration and remote thermal infrared observation revealed that a large fraction of Solar System small bodies should be covered with granular regolith. The complex and varied geology of the regolith layer may preserve the historical records of the surface modification and topographic evolution experienced by asteroids, especially cratering processes, in which the projectile shape plays a crucial role. Regarding the impact sampling scheme, the projectile-shape dependence of both the cavity morphology and the collected mass remains to be explored. This paper studies the process of the low-speed impact sampling on granular regolith using projectiles of different shapes. The results demonstrate that the projectile shape significantly influences the excavation stage, forming cavities with different morphologies, i.e., cone-shaped, bowl-shaped and U-shaped. We further indicate that the different velocity distributions of the ejecta curtains due to the various projectile shapes result in various amounts of collected mass in sampler canister, regarding which the 60° conical projectile exhibits preferable performance for impact sampling scheme. The results presented in this article are expected to reveal the dependence of the excavation process on projectile shape under micro gravity and provide further information on the optimal designs of impact sampling devices for future sample-return space missions.

## Introduction

Space missions and remote thermal infrared observations have shown that the surfaces of most asteroids are covered with regolith, a layer of granular materials that has a size distribution ranging from very fine dust^1, 2^, to coarser gravel-like grains^3, 4^. The complex geology of the regolith layer may preserve the records of the surface modification experienced by asteroids^5–7^. Additionally, organic matter and hydrated minerals within the regolith are expected to provide crucial information on the origin and evolution of the planets, especially the source of organic molecules and water, which has recently attracted the attention of different international space agencies, e.g., NASA with OSIRIS-REx^4^ and JAXA with Hayabusa-2^8^. China is also planning to develop an asteroid exploration mission with surface impact sampling included^9^. Within the Chinese space program, then, understanding the impact dynamics related to granular regolith facilitate the development of efficient anchoring tools or impact sampling mechanism designs^10^ for sample-return space missions. In addition to the technology perspective, insight into impact dynamics is also useful to broaden our interpretation of the physical properties^11^ and surface geology^12^, including crater morphology, of these celestial objects in the Solar System.

Although laboratory experiments have provided much insight into cratering phenomena in terms of qualitative descriptions or semi-analytic expressions, including bulb-shaped tracks on porous targets^13^, crater-ray formations of impact ejecta^14^, and low-speed penetration resistance^15–17^, coupled with granular flow and compaction^18, 19^, we still do not fully understand the cratering process, partially due to the difficulties in experimentally tracing the motions of target particles. Such limitation can be overcome through numerical simulations, e.g., by using the Soft-Sphere Discrete Element Method developed by Cundall & Strack^10, 20^, which consequently complements our understanding of the excavation stage^12, 21^, and the amount of ejected mass^22, 23^. However, the dependence of cavity morphology and collected mass on the projectile shape remains to be explored, which is essential information for impact sampling mechanism design.

In this work, we numerically study the process of the low-speed impact sampling of asteroid granular regolith, focusing on how the projectile shape determines the excavation stage and ejection flow. We carry out systematic simulations to find out the dependence of the cavity morphology and penetration regime on the projectile radius and sharpness, by using cones of various angles, hemispheres and disks as projectile geometry (see Table 1). Fixed the projectile mass (≈4.19 g), the projectile shape that tends to produce the greatest amount of ejected materials (collected by sample catcher canister within limited sampling time) is identified.

**Table 1:** Shape parameters of various projectiles and the corresponding inertial drag coefficients, coupled with the maximum energy of granular targets.

No.	Sharpness	Radius [cm]	*C*_*d*_	Energy [mJ]	No.	Sharpness	Radius [cm]	*C*_*d*_	Energy [mJ]
1	Disks	0.35	5.64	201	16	90° cones	0.35	3.04	161
2		0.50	5.84	221	17		0.50	3.13	199
3		0.55	5.97	302	18		0.55	3.21	217
4		0.60	5.94	371	19		0.60	3.24	241
5		R* = 0.69	5.92	361	20		R* = 0.79	3.00	274
6	Hemispheres	0.35	3.75	158	21	120° cones	0.35	4.57	211
7		0.50	3.73	208	22		0.50	4.50	223
8		0.55	3.75	226	23		0.55	4.56	249
9		0.60	3.82	240	24		0.60	4.72	291
10		R* = 0.63	4.09	247	25		R* = 0.95	3.13	358
11	60° cones	0.35	1.51	128	3^+^	Disks	0.55	5.91	312
12		0.50	1.55	189	8^+^	Hemispheres	0.55	3.72	228
13		0.55	1.62	197	13^+^	60° cones	0.55	1.66	194
14		0.60	1.54	213	18^+^	90° cones	0.55	3.03	219
15		R* = 0.66	1.46	223	23^+^	120° cones	0.55	4.46	255

Twenty-five projectiles with cylindrical tails and various-shaped noses are used in the simulations, including disks, hemispheres, 60° cones, 90° cones and 120° cones, whereas R* corresponds to projectiles without tails. However, the granular particles are never in contact with the tail; hence, the tail’s presence is unrelated to drag resistance, whereas only increasing the mass of the projectile and therefore keeping mass equal with different shaped projectiles. As an additional simulation suite, five more impact scenarios (denoted by+), with various impact points within a 1-mm circular region, are carried out, revealing no discernible effect on the cavity morphology and collected mass.

## Results

**Figure 1:**
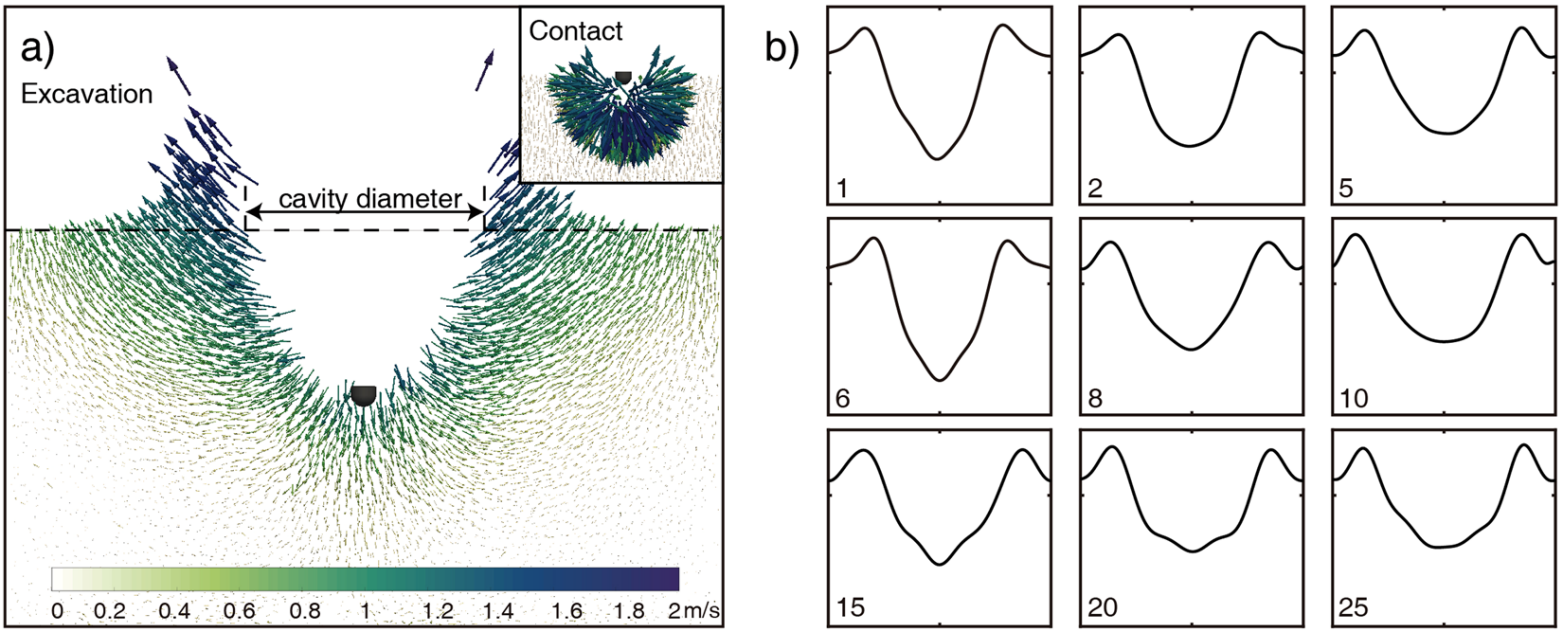
**(a)** Velocity vector field of the cross-sectional view in the contact stage and excavation stage (case Sim. 8 in Table 1). The projectile slightly penetrates into the surface of the granular target immediately after impact, subsequently shoving away target particles and forming an excavation cavity. The insert view shows the steep rise in particle velocity with the distance from the contact point. The scale color for velocity is shown at the bottom. **(b)** Cavity morphology of various projectile shapes. Blunt projectiles yield bowl-shaped cavities (Sim. 5, Sim. 25), whereas pointed projectiles yield cone-shaped cavities (Sim. 1, Sim. 6); projectiles in between generate cavities more like U-shaped (Sim. 2). Each subgraph corresponds to a specific projectile in Table 1.

Twenty-five projectiles with different shapes and the same mass are propelled above a granular target, which serves as a reasonable analog of coarse-gravel asteroid regolith, and allowed to impact vertically into it at a velocity of 25 m s^−1^. These projectiles subsequently generate excavation cavities of various morphologies, resulting in entirely different ejecta curtains and different amounts of mass collected by the sampler mechanism, which is set to rest on the regolith surface throughout the sampling. In our simulation, this impact process is divided into two distinct regimes: the contact stage, from impact until fragmentation of the granular bed, and the excavation stage, the period of intrusion of the projectiles^21^, as illustrated in Fig. 1a.

The projectile slightly penetrates into the surface of the granular target in the regime immediately after impact, characterized by mild contact but appreciable compression. The target grains seem nearly at rest but actually have prodigious velocities, which results in the formation of the steep rise in particle velocity with the distance from the impact point (Fig. 1a). Due to the loose and discontinuous nature of the granular target, the impulsive force is transmitted into the material via compression pulses in the vicinity of the projectile, yielding spatially sparse, heterogeneous force chains^15, 19, 24^. As the projectile penetrates into the granular bed, target particles are forcefully displaced due to high-velocity intrusion, consequently generating forced excavation flows. During this process, the cavity opens and expands with radial growth, as observed in laboratory experiments and numerical simulations^12, 15^, forming excavation cavities of various morphologies. Meanwhile, individual force chains exceed the plastic yield strength and subsequently break down on account of the drastic impact action adjacent to the projectile tip, transmitting most of the initial kinetic energy to the granular particles. Such a drastic transmission of energy also releases particles from the top surface of the granular bed, forming nearly inverse conical-shaped ejecta curtains^14, 23, 25^. Subsequently, ejected particles with different velocity distributions are concentrated through the conical horn against the force of the asteroid gravity until moving up into the catcher canister^26^, the top of the sampler similar to Hayabusa^26^, yielding different amounts of collected mass. Due to to the longer ejecta timescales involved under low-gravity conditions^22^, we did not find any inward flow, which means gravitational collapse as observed experimentally^27, 28^, throughout the entire sampling process. Thus, in this study, a transient cavity is simply assumed at the moment the projectiles drop below one ten thousandth of their incident kinetic energy. Such definition ensures that the subsequent expansion of the cavity is sufficiently small, which makes the transient cavity at this time typical for analyzing excavation characteristics of various shaped projectiles.

In the following subsections, we will focus on the projectile-shape dependence of cavity morphology and collected mass in detail.

### Cavity morphology

As illustrated in Fig. 1b, cavity morphology varies significantly with projectile shape, especially with the sharpness and radius. Blunt projectiles excavate bowl-shaped cavities with a relatively shallow depth but large radius (Sim. 5, Sim. 25), in which case lateral dilation-induced cavitation predominantly occurs due to the larger horizontal velocity^29^. Accordingly, the lateral displacement and volumetric strain of such cavities are relatively large. In contrast, the cavities produced by pointed projectiles, i.e., cone-shaped cavities (Sim. 1, Sim. 6), are characterized by deeper but narrower penetration tracks in conjunction with stress concentration immediately below the projectile tip^30^. Additionally, the embedded regions generated by the vigorous penetration of pointed projectiles (e.g., Sim. 15, which corresponds to a 60° cone) match up well with the result of previous experiments^29, 30^, whereas those of blunt projectiles are not discernible in our simulation. Furthermore, projectiles with shapes intermediate between blunt and pointed generate U-shaped cavities (Sim. 2) with medium-sized radii and average penetrations.

**Figure 2:**
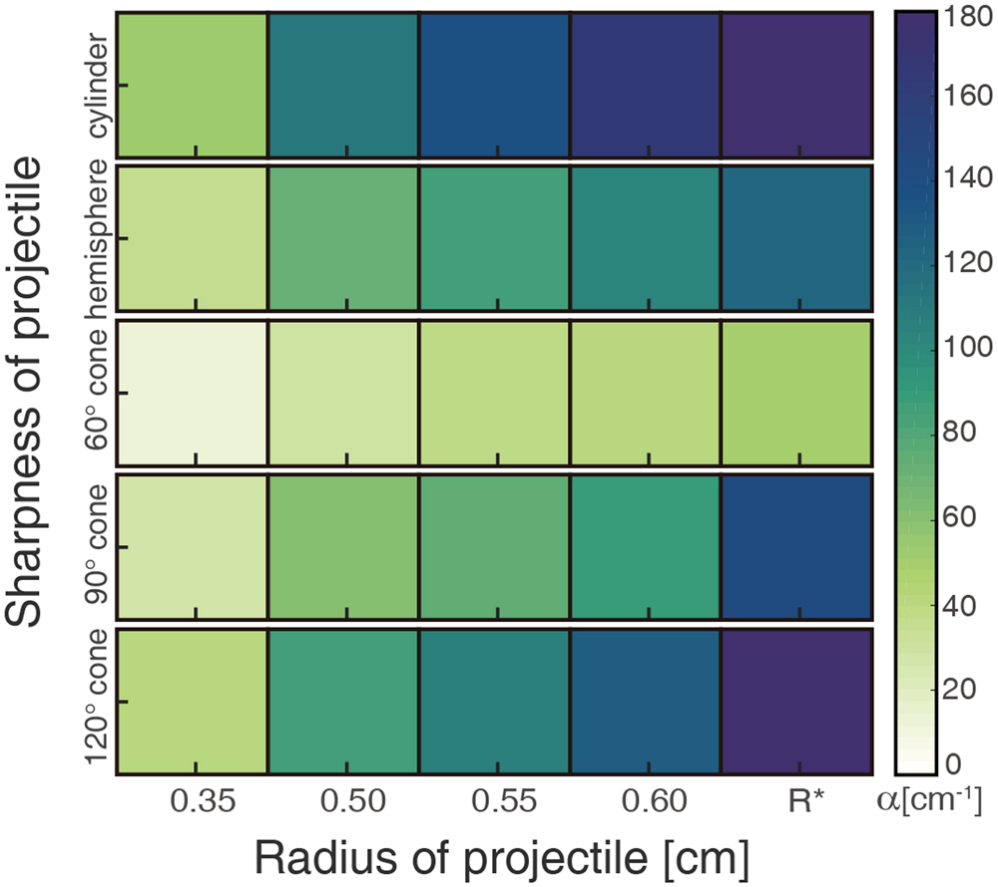
The equivalent resistance coefficients *α* of various shaped projectiles. Each rectangular region represents a specific combination of sharpness and radius, colored based on the equivalent resistance coefficients *α* according to the color legend to the right of the plot. For all simulations, an increase in the projectile radius systematically increases the intrusion resistance regardless of the tip sharpness (denoted by a change in color from light to dark). The opposite is true regarding the projectile sharpness, which shows an anti-correlation characterized by greater equivalent resistance coefficients as projectiles become more blunt, i.e., cylinder >120° cone > hemisphere > 90° cone > 60° cone. Note that the values of correlation coefficient for all fitting curves are higher than 0.985.

To gain greater insight into the excavation dynamics, it is necessary to understand how the interaction between the projectile and the granular bed changes with penetration, which reflects the features of the projectile shape indirectly. Previous studies^15–17, 31^, have demonstrated that the resistance of a granular medium can be considered as the sum of the hydrodynamic inertial drag, the viscous-like drag and the hydrostatic-like force, which is given by

1 $${M}_{p}\frac{dv}{dt}=-(\frac{1}{2}{C}_{d}{\rho }_{t}{S}_{p}{v}^{2}+6\pi \eta {R}_{p}v+{F}_{0})$$

where *ρ*_*t*_ is the bulk density of the target and *M*_*p*_, *R*_*p*_, *S*_*p*_, *C*_*d*_ are the mass, radius, cross-section area and resistance coefficient of the projectile, respectively. The second term on the right-hand side, 6*πηR*_*p*_ *ν*, represents viscous-like drag, in which *η* corresponds to only the frictional character of granular materials^15^ and hence is determined at approximately 25.0 Pa s via data fitting (greater than the value used in granular impact experiments^15^ but smaller than that used in flow-induced granular agitations^32^) for all simulations. Additionally, the velocity-independent resistance force, *F*_0_, which is proportional to the gravitational acceleration and granular medium porosity, is ignored considering that it has no discernible effect due to the low-gravity on asteroids. Consequently, the equivalent resistance coefficients, $\alpha =\frac{1}{2}{C}_{d}{\rho }_{t}{S}_{p}/{M}_{p}$, determined via discrete data-fitting in high-velocity regime (*v* > 1.0 m s^−1^) are as shown in Fig. 2.

It is observable that there is significant variation in the equivalent resistance coefficients *α* between different projectile shapes. An increasing in the projectile radius systematically increases the intrusion resistance force according to the square law, consequently reducing the penetration depth regardless of the tip sharpness, which is consistent with the results of previous low-speed impact experiments^17, 33^. Additionally, projectile sharpness also plays a crucial role in the deceleration process, creating a scaling dependence, with the equivalent resistance coefficients decreasing as the sharpness increases, i.e., 60° cone < 90° cone < hemisphere < 120° cone < cylinder. Such features match up well with the phenomena observed in previous experiments^29,31,33, 34^, and with that shown in Fig. 1. Furthermore, the inertial drag coefficients *C*_*d*_ derived from the penetration motion of projectiles with the same sharpness show reasonable conformance for various radii (Table 1), suggesting that *C*_*d*_ could describe the dynamical characteristics of pointed or blunt projectiles effectively, which allows us to confidently extend the force law of spheres to non-spherical projectiles. Such extension may improve our understanding of impact craters in asteroid regolith caused by cuspidal or obtuse projectiles.

### Collected mass

**Figure 3:**
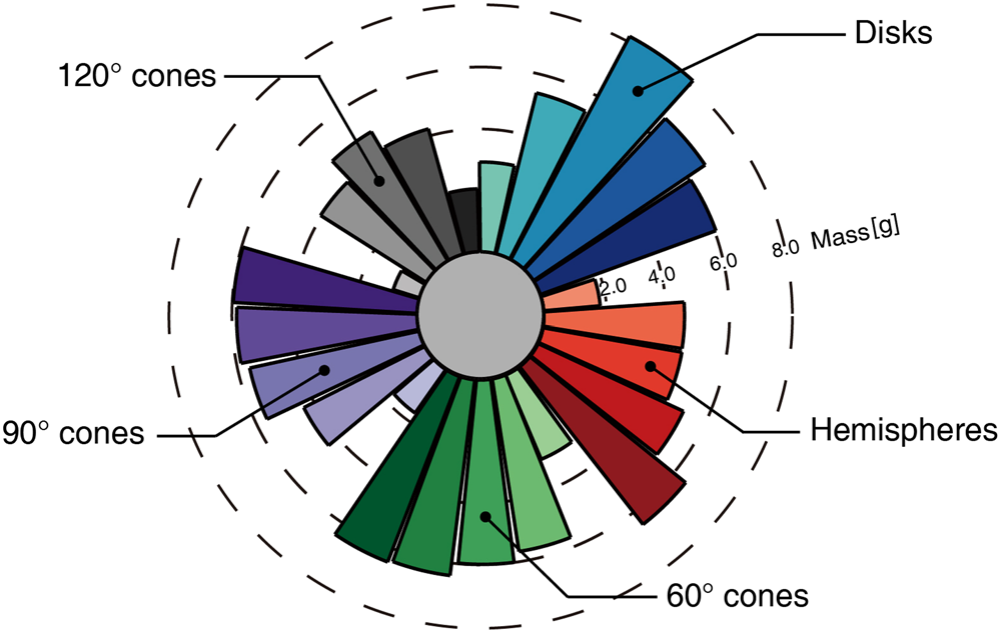
Collected mass in sampler canisters within 1s sampling time of various projectile shapes. The figure shows the collected mass in association with the impact of projectiles with the following shapes: disk (blue), hemisphere (red), 60° cone (green), 90° cone (purple), 120° cone (gray), with radii ranging from 0.35 cm to R* (colors ranging from light to dark, respectively) as shown in Table 1.

The ejecta mass collected by the sampling canister varies significantly with the projectile shape, particularly with the sharpness and the radius. As Fig. 3 demonstrates, the collected mass is defined as the amount of ejected material collected by the canister within a limited sampling time, i.e., the amount of grains expelled into the catcher canister, the top of the sampler (see Methods section), after a given time is calculated to determine the collected mass. In summary, the collected mass of pointed projectiles, such as the 60° cone or 90° cone, consistently increases with the increase of the cross-sectional area, whereas that of blunt projectiles decreases after ascent, with an extremum forming for medium-sized radii. We speculate that the energy transmission, in terms of the interaction between projectiles and granular particles, is proportional to the inertial drag resistance and hence the projectile radius, as shown in Table 1, leading to the positive correlation between collected mass and projectile radius. In contrast, blunt projectiles, e.g., the 120° cone or cylinder, have resistance coefficients that are too large, preventing them from penetrating the target with an overly large radius; thus, less materials are ejected, subsequently reducing the collected mass, suggesting that a medium-sized radius may be an appropriate choice when using disks or obtuse cones for impact sampling mechanism.

**Figure 4:**
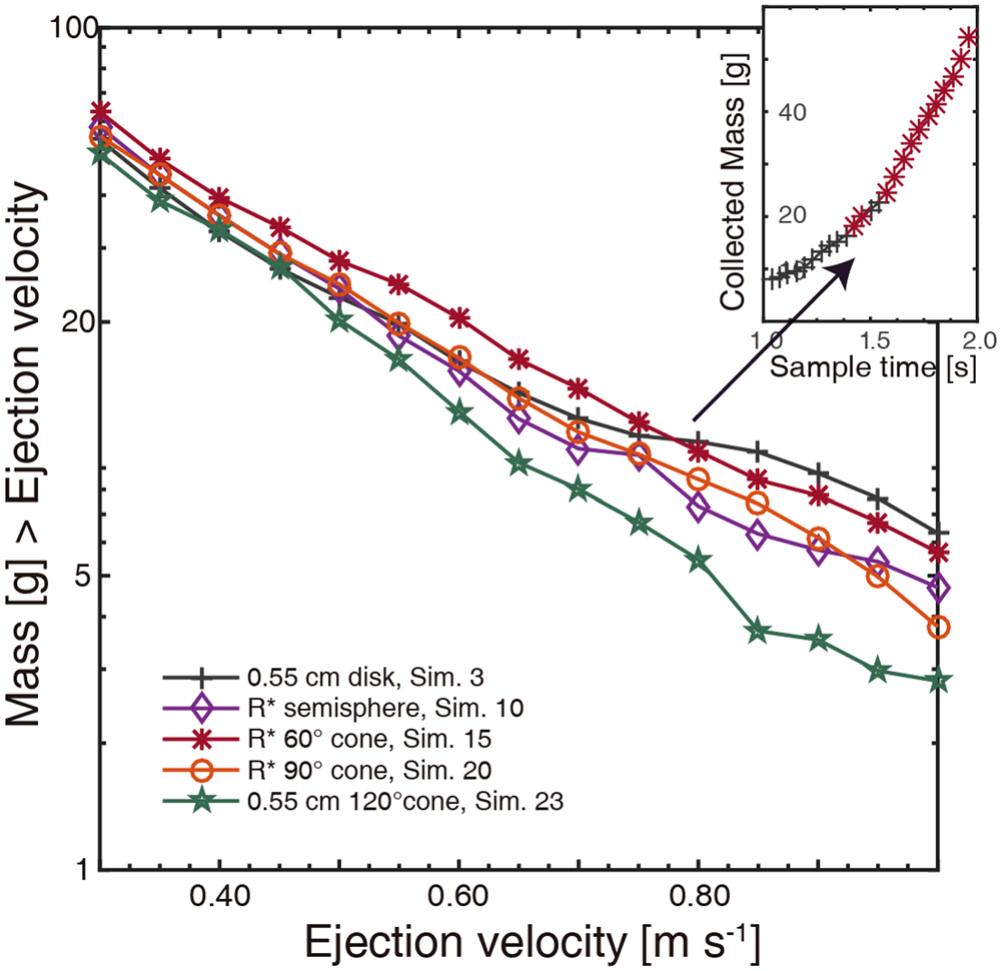
Velocity spectra of the ejected particles resulting from various shaped projectiles with the optimal radius. The relative order varies significantly from high velocity to low velocity, revealing that the 0.55 cm cylinder ejects more gravels in relatively short sampling time (indicating higher *v*_eff_), whereas the 60° cone has superior performance for long sampling time (indicating lower *v*_eff_), which is consistent with the sampling outcomes in various sampling times showed in insert. Insert. Optimal collected mass versus the sampling time. The changing point of the optimal projectile shape corresponds to the crossover point of the cumulative velocity distributions.

The distribution of cumulative mass versus the ejection velocity of the ejecta curtain, i.e., the velocity spectra, is a key factor that determines the amount of collected mass. Accordingly, the ejection velocity distributions, in which the ejection velocity is defined as the vertical velocity when passing through the regolith target surface plane, are analyzed to characterize the source of variation in collected mass between different projectiles. Figure 4 illustrates the cumulative velocity distributions of projectiles of various sharpness with the optimal radius, i.e., the one generating the largest amount of collected mass among the five radii (Table. 1), showing significant differences with each other. It is notable that only grains with vertical velocity exceeding a certain critical velocity, i.e., the effective velocity, can be collected by the sampler canister due to the limited sampling time. Consequently, such effective velocity is closely related to the actual sampling time and can be roughly determined by ${v}_{{\rm{eff}}}=\lambda H/{T}_{{\rm{sample}}}$, where *H*, *T*_sample_ are the distance from the target surface to the sampler canister and the sampling time, respectively. The dimensionless parameter *λ* (>1) reflects the deceleration of the vertical motion during each grain-sampler collision. Therefore, only grains with an ejection velocity exceeding the effective velocity *v*_eff_ yield the effective ejected mass for a given sampling time *T*_sample_. Consequently, the short-term sampling would give a higher effective velocity compared to that of long time sampling, in which case the projectile generating larger amount of ejecta with high ejection velocity would have better performance in impact sampling. In contrast, in the case of a relatively long time sampling, grains with a lower ejection velocity would still have the possibility to be collected due to the lower effective velocity. As observed in Fig. 4, the 0.55 cm cylindrical projectile (Sim. 3) produces more ejecta with high velocity but less ejecta with relatively low velocity compared to the 60° conical projectile, consequently generating larger amount of collected mass in the short sampling time (indicating the higher effective velocity), whereas the 60° conical projectile (Sim. 15) has better performance in relative long sampling time (indicating the lower effective velocity). Such variation tendency is approximately consistent with the sampling outcome in various sampling times (Figs 3, 4), suggesting that the combination of the ejecta velocity spectrum and the effective velocity could be used to predict the dependence of the collected mass on the projectile shape quite well.

Furthermore, it is significant to note that our result, i.e., that 60° conical projectiles appear to eject the largest amount of collected mass in relatively long sampling time, seems to be different from the experimental findings of Makabe and Yano^35^, which indicated that 90° conical projectiles have the most suitable shape for the impact sampling mechanism. We speculate that this disagreement may come from the narrow range of projectile shapes explored in their laboratory experiments. For example, the radius of the 60° cone used by Makabe and Yano was only 0.50 cm (corresponding to Sim. 12 in our simulations), which is less than the optimal radius R* of 60° cones determined based on the positive correlation between collected mass and projectile radius (Fig. 3). Additionally, a more realistic sampling process would include sampler mechanism modeling. This modeling was removed due to the limitations of the experimental setup^26^, which may have caused the difference. Nevertheless, we can conclude that the 60° conical projectiles have preferable performance according to our research, especially for the relatively long-term impact sampling process.

## Discussion

In the present study, we demonstrate that the projectile shape, especially the sharpness and the radius, significantly influences the excavation stage and ejecta flow, forming cavities with different morphologies, i.e., blunt projectiles excavate bowl-shaped cavities whereas pointed projectiles generate cone-shaped cavities. We further indicate that the different velocity distributions of the ejecta curtains due to the various projectile shapes, lead to variation in collected mass of sampler canister. The collected mass of pointed projectiles consistently increases with the increase of the cross-sectional area, while that of blunt projectiles first ascends and then descends, forming an extremum for medium-sized radii. The 60° conical projectiles have preferable performance, especially for the relatively long-term impact sampling scheme.

Furthermore, the inertial drag coefficient *C*_*d*_ seems to be closely correlated with the sharpness factor, *I* = 1/sin^2^ *θ*, where *θ* is half of the taper angle of projectiles. This correlation indicates that the inertial drag resistance should originate from the perpendicular and intermittent impacts of projectiles with the target grains^31, 36^. Hence, the total drag force can be calculated by taking the integral of the individual momentum-transfer induced force over the surface area, expressed as

2 $$F=\int f\frac{dS}{{{r}_{t}}^{2}}\propto \int \frac{{m}_{t}{v}^{2}{\sin }^{2}\theta }{{{r}_{t}}^{3}}dS\propto {\sin }^{2}\theta {\rho }_{t}{S}_{p}{v}^{2},$$

which yields ${C}_{d}\propto {\sin }^{2}\theta ={I}^{-1}$, as observed in the fitting results, where *m*_*t*_, *r*_*t*_ are the target particle mass and radius, respectively. Accordingly, the inertial drag coefficients of various sharpness are unified through connecting the microscopic grain-scale physical processes to the macroscopic force law, which may facilitate the detection of the subsurface structure of small bodies via contact measurements from on-board instruments^37, 38^. However, such microscopic explanation regarding the difference in collected mass between various projectile shapes still requires further study, e.g., the spatial structure of heterogeneous force chains^31^ and nonlinear force propagations^19^.

In general, we believe the results presented in this paper reveal the projectile-shape dependence of cavity morphology and collected mass, which is expected to broaden our understanding of the excavation processes that occurred during the dynamical evolution of celestial body surfaces in the Solar System and as well to benefit the design of efficient impact sampling devices on asteroid regolith for future sample-return space missions.

## Methods

### DEM

In this study, an effective, parallel, three-dimensional N-body numerical code, Discrete Element Method (DEM)^10, 20, 39^, in which elastic spherical particles (i.e., soft-sphere) are described by linear-spring dashpot in conjunction with slider model, is carried out to simulate the impact process of various projectiles into granular regolith based on our previous work^40^. The motion of each particles is simultaneously calculated by taking into account whole mechanical interactions that occur when particles make contact, such as various kinds of friction, including rolling friction, which corresponds to the transformation of rotation energy into friction energy, and the normal and tangential deformation of colliding particles. The resultant contact forces on particles are given by

3 $$\begin{array}{l}{F}_{n}=-{k}_{n}x+{C}_{n}{u}_{n}+{F}_{c},\\ {F}_{t}=\,\min \,\{{k}_{t}S+{C}_{t}{u}_{t},{\mu }_{s}|{F}_{n}|\},\end{array}$$

which depend on the spring constants, *k*_*n*_ and *k*_*t*_, the plastic damping parameters, *C*_*n*_ and *C*_*t*_ (which are related to the normal and tangential coefficients of restitution, *ε*_*n*_ and *ε*_*t*_, respectively), and the dimensionless coefficients *μ*_*s*_ providing an effective stick-slip friction between colliding particles, respectively. The variable *x* indicates the mutual compression of contacting particles, and *S* is the total tangential elongation that occurs during collision. The dashpot forces are linearly proportional to the relative velocity components, *u*_*n*_ and *u*_*t*_. Furthermore, the rolling friction torque model ${M}_{r}=-{\mu }_{r}|{F}_{n}|{R}_{i}{\hat{\omega }}_{i}$, determined by the rolling friction coefficient *μ*_*r*_, the particle radius *R*_*i*_ and normalized angular velocity ${\hat{\omega }}_{i}$, in our code is improved based on numerous considerable debates in the materials science; this model has been fully validated through comparison with laboratory experiments and successful applications^41–43^. Additionally, the van der Waals force can become relevant in such a low-gravity regimes^44–47^, considered in our simulation; thus a simple dry cohesion model ${F}_{c}={A}_{h}{R}_{i}{R}_{j}/({R}_{i}+{R}_{j})$ is adopted to mimic the weak attractive inter-particle force in asteroid regolith, where ${A}_{h}=0.036\,{\rm{N}}\,{{\rm{m}}}^{-{\rm{1}}}$, obtained through a comprehensive investigation of lunar regolith^48^, is used as a representative value throughout the study.

Although very little is known about the actual mechanical properties of asteroid regolith, the constituent particles must be frictional, i.e., a coarse-grained surface on the small bodies. To learn more regarding the impact process, a more realistic set of soft-sphere parameters (Table 2) is adopted to appropriately reflect the typical behaviors of the gravel. These parameters are similar to the ‘gravel’ parameters verified by comparing series of simulations with avalanche experiments using coarse rocks^49^, in which the large *μ*_*s*_, *μ*_*r*_ and small *ε*_*n*_, *ε*_*t*_ account for the irregular non-spherical shapes of the real granular matter^50, 51^.

**Table 2:** Numerical values of the parameters used in the simulations. The same values for the spring constants, the coefficients of restitution and the friction are used to govern each collision, i.e., grain-grain, grain-projectile, grain-sampler, and grain-container. Based on the sample analysis of Itokawa’s regolith, the density of the target materials ρ_*t*_ is set to 3.2 g cm^−3^. In addition, all projectiles are made of SUS304 metal for consistency with the experimental setting used by Makabe and Yano^35^.

*k*_*n*_[kg/s^2^]	*k*_*t*_[kg/s^2^]	*ε*_*n*_	*ε*_*t*_	*μ*_s_	*μ*_*r*_	${\rho }_{t}\,[{g/\mathrm{cm}}^{{\rm{3}}}]$	${\rho }_{p}\,[{g/\mathrm{cm}}^{{\rm{3}}}]$
1.05×10^7^	2.99×10^6^	0.55	0.55	0.81	3.0	3.2	8.0

### Granular regolith

The granular regolith in the simulation consists of 100,854 spherical particles with radii drawn from a size distribution with a mean of 0.22 cm and with truncated widths of ±0.03 cm, consistent with that of Itokawa’s regolith, which was constrained via close surface observations and samples analysis^26, 53, 54^. To further avoid crystallization of the granular configuration, we randomly drop these particles, with small and stochastic initial velocity, into an empty cylindrical container, with diameter of 26.0 cm and height of 16.0 cm, under the influence of the asteroid’s gravity, i.e., 10 microG in our simulations, a typical surface gravity on small asteroids. We then subject the container to forced slight oscillations along the axis of the cylinder, disorganizing the possible artificial shear strength structure^51^, which we then allow to resettle inside. After ensuring that all motion has already ceased, we find that the granular regolith ends up with a bulk porosity of ~42%, similar to the estimated macroporosity of radar-detected C-class or S-class asteroids^4, 55^, and to the precisely measured near-surface bulk porosity of the asteroid Itokawa (~41%)^54^.

### Combined walls

**Figure 5:**
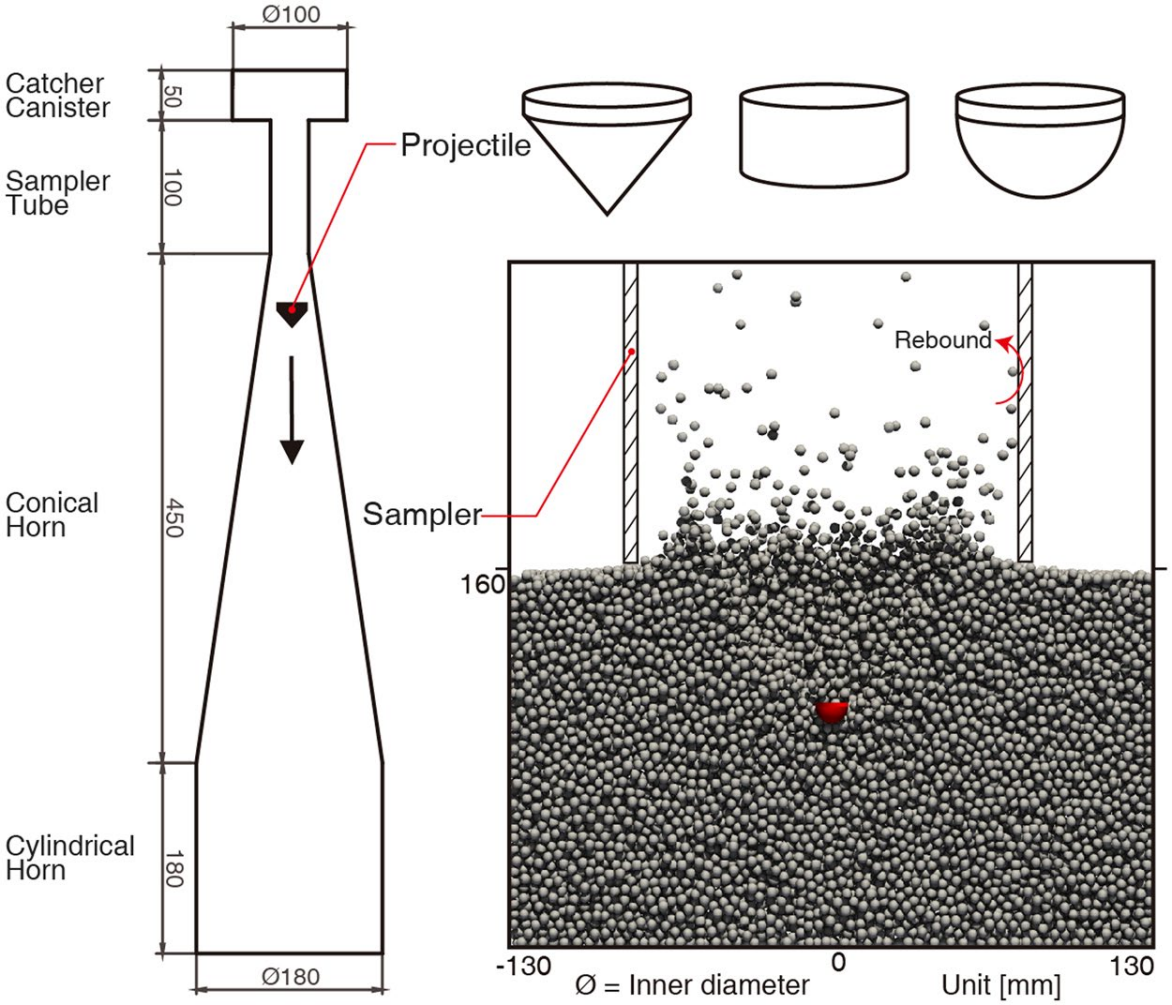
Complex-shaped sampler and projectiles. The sampler rests on the regolith surface throughout the sampling, which consists of a cylindrical horn, conical horn, sampler tube and catcher canister, similar to that of Hayabusa^26, 52^. Combined walls are jointed to constitute the complex shapes, e.g., conical projectiles are formed with a sharp nose point, conical surface, intersecting circle, cylindrical surface, intersecting circle and circular surface from bottom to top. The walls in different geometries are applied in the detection of the point-sphere, surface-sphere, and edge-sphere contacts, respectively.

Motivated by simulating the collision between grains and complex-shaped sampler or projectiles, an additional well-tested method developed by Schwartz *et al*.^22^, is adapted to account for the effects of non-spherical objects by using different combinations of arbitrary numbers of movable walls, consisting of sharp nose point, intersecting circle, conical surface, cylindrical surface, etc. (Fig. 5). To simplify the computational complexity, the projectiles formed by the combined walls only feel resistance from the granular target along the direction of the initial trajectory of projectiles, which means that the slight yawing rotation and translational motion perpendicular to the direction of the velocity are ignored^22^, considering that the momentum transferred to projectiles along the direction of their initial trajectory dominates the majority of the total interaction with the regolith. Additionally, the rotation or lateral motion of the projectiles is not very significant during penetration into the granular regolith, thus having an extremely slight influence on their motion, as deduced from the extremely symmetrical ejecta curtain observed in the experiment^35^.

### Comparison to impact experiments

**Figure 6:**
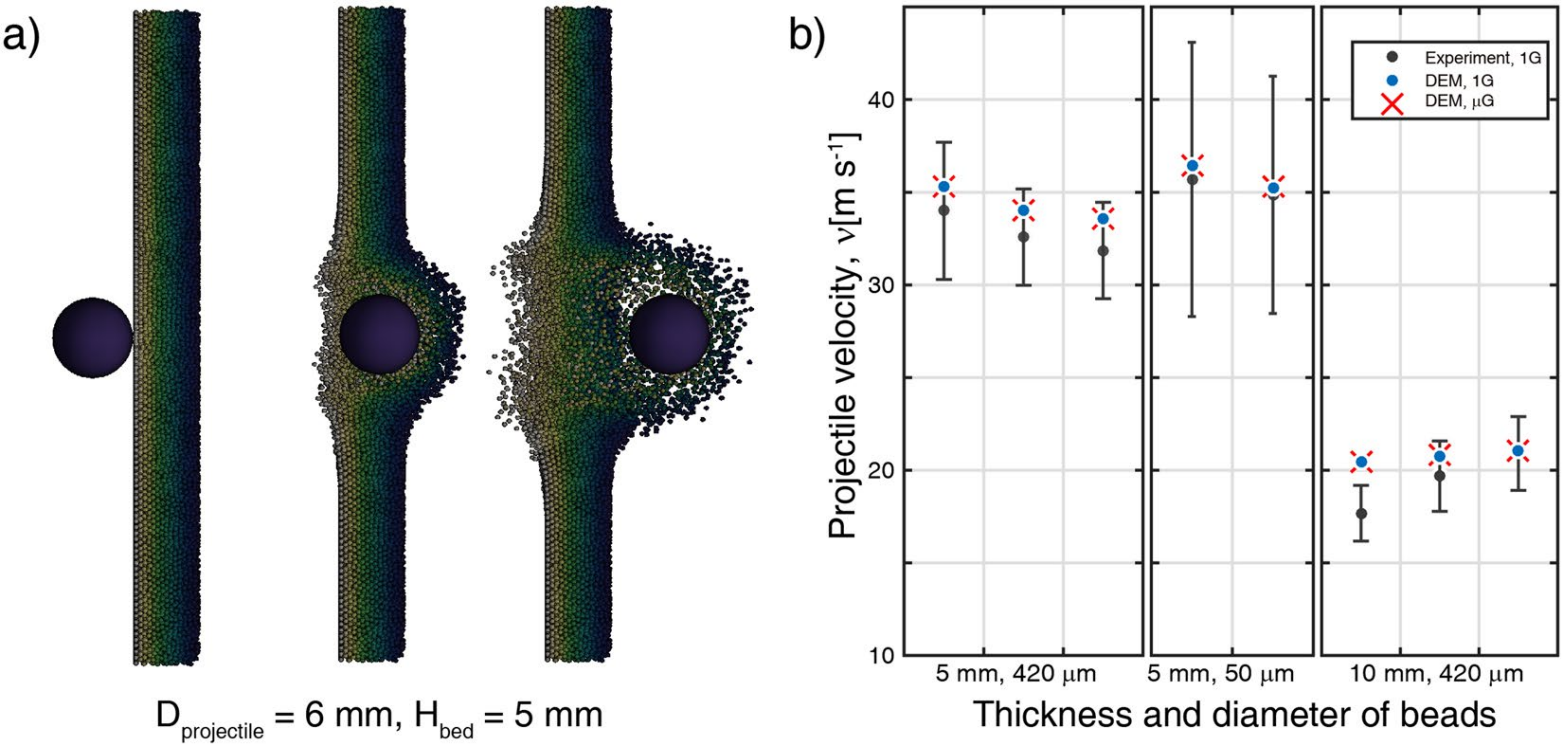
(**a**) Half-sectional views of projectile penetrating a granular bed. A plastic spherical projectile with a diameter of 6 mm penetrates through the granular target with a thickness of 5 mm. These frames are presented in chronological order. (**b**) Projectile velocity after penetration of glass beads. The simulations under 1G (blue) and *μ*G (red) show reasonable consistence with the experiments (black) within the uncertainty of the measurements.

To demonstrate whether our numerical code can be used to model the impact and intrusion process due to low-velocity impacts into granular matter, we then simulate a laboratory experiment using a plastic sphere penetrating through granular beds with different thicknesses at a velocity of 67.5–70.1 m s^−1^. The granular beds were packed with polydisperse soda lime glass beads with an average diameter of either 50 *μ*m or 420 *μ*m^15^, which were supported by acrylic plates covered by a very thin layer of paper and aluminum foil on each side (see ref. 14. for a detailed description). The numerical results show that the post-penetration velocity has no obvious relevance to the gravity conditions but systematically decreases as either the granular layer thickness or the glass bead diameter increases, as observed in the experiments. In each case, we find a satisfying agreement with the experimental outcomes within the uncertainty of the measurements (Fig. 6). In summary, this verification and those in Supplementary Information allow us to confidently apply our numerical code to low-velocity impact simulations, especially for the analysis of the excavation processes and the ejecta curtains that occurred during the dynamical evolution of celestial body surfaces in the Solar System or the analysis of the impact sampling processes conducted on asteroid regolith during sample-return space missions, which is the major subject of this research.

### Data Availability

The datasets generated during and/or analysed during the current study are available from the corresponding author on reasonable request.

## Electronic supplementary material

Supplementary Information
