# Supplementary material for: Asteroid surface impact sampling: dependence of the cavity morphology and collected mass on projectile shape
Source: Sci Rep. 2017 Aug 30;7:10004. doi: 10.1038/s41598-017-10681-8 (PMC5577283; doi:10.1038/s41598-017-10681-8)
Supplement: Supplementary file 1 — Supplementary Information [file 41598_2017_10681_MOESM1_ESM.pdf]

Supplementary Information

Asteroid surface impact sampling: dependence of the cavity morphology and collected mass on projectile shape

*Bin Cheng[1], Yang Yu[2], and Hexi Baoyin[1]**

[1] Tsinghua University, Beijing, 100084, China

[2] Beihang University, Beijing, 100191, China

* Correspondence should be addressed to: baoyin@tsinghua.edu.cn

To demonstrate whether our code can be used to model the impact and intrusion process due to low-velocity impacts into granular matter, we then perform a series of simulations to reproduce the results of various experiments covering a broad velocity range, i.e., the stagnant zone formation in unsteady hopper flow (~0.1 m s^{-1}), the ejected mass of the impacting process (~1 m s^{-1}), and the scaling law of the crater morphology (~10 m s^{-1}). In the following sections, we will report on these tests designed to demonstrate correct dynamic behavior of the granular material in details.

Stagnant zone

A sandpile can be formed inside a container after discharging particles, giving the so-called stagnant zone in unsteady hopper flow[1]. We then simulate a laboratory experiment, with a rectangular container packed by soda lime glass beads with an average diameter of either 10 mm or 6 mm. The simulation settings, e.g., the geometry of the container, the model parameters and the simulated procedure, are the same as those used in the experiments[2] (see Ref. [2] for a detailed description). As illustrated in Fig. S1, the simulation is started with the random generation of spheres in the container, followed with a gravitational settling process to form a stable packing, which is then used as the initial condition for discharging. Afterwards, the instantaneous opening of the outlets starts a discharging process in which spheres flow into the bottom container under the gravity, whereas some spheres remains on the central plate, forming a stable stagnant zone as shown in Fig. S1. Obviously, the simulated morphologies of the stagnant zone are quite comparable with the measurements for the two sphere sizes, respectively. Additionally, the number of spheres remaining on the central plate of repeated simulations is 118–143 for 10 mm spheres, and 771–823 for 6 mm spheres, which show a satisfying agreement with the experimental outcomes that the number of spheres in stagnant zone is 128±3 for 10 mm and 786±11 for 6 mm spheres[2], respectively. Comparison between the simulated and experimental results under comparable conditions confirms the validity of the code used in this study.

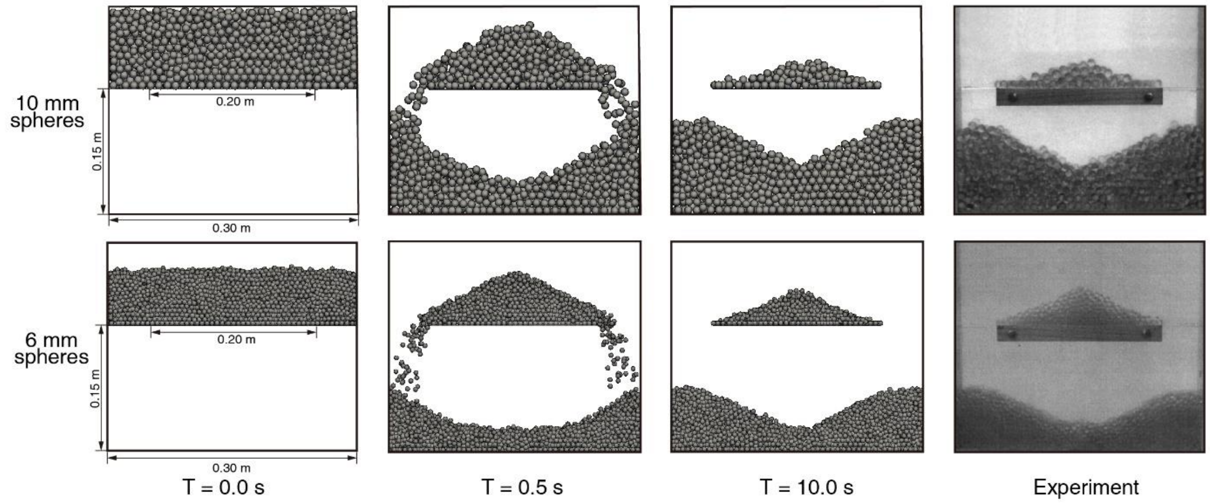

Supplementary Figure S1: Snapshots showing the formation of the stagnant zone for 10 mm spheres and 6 mm spheres at different discharge times. The morphologies of the stagnant zone show a satisfied agreement with the experimental outcomes[2].

Ejected mass

Motivated by the development of safe and effective techniques for operations at the surface of celestial objects, the ejected mass-velocity distribution of low-speed impacts during these regolith-spacecraft interactions was investigated experimentally[3]. Thus we numerically reproduce this experiment under the same conditions, i.e., using a 19.71 mm glass projectile vertically impacting into a granular bed of 2 mm acrylic beads (see Ref. [3] for a detailed description). We perform a total of thirty simulations using a range of the impact velocity (0.9–3.6 m s^{-1}) and height of the granular bed (46.1–138.3 mm), and then study the changes in the ejected mass-velocity distribution for different impacts, in which the ejected mass is determined by the difference between the initial and final particle masses in the container. As illustrated in Fig. S2, the numerical results show that the ejected mass systematically increases as the impact velocity increases. The opposite is true regarding the height of the granular bed, which shows an anti-correlation characterized by less amount of ejected mass as the granular height become larger. Additionally, in each case, we find a good accordance for the total ejecta mass with the experimental results within the 95% confidence interval of the experiment data, validating the numerical code for predicting the outcomes of low-velocity impacts into granular media.

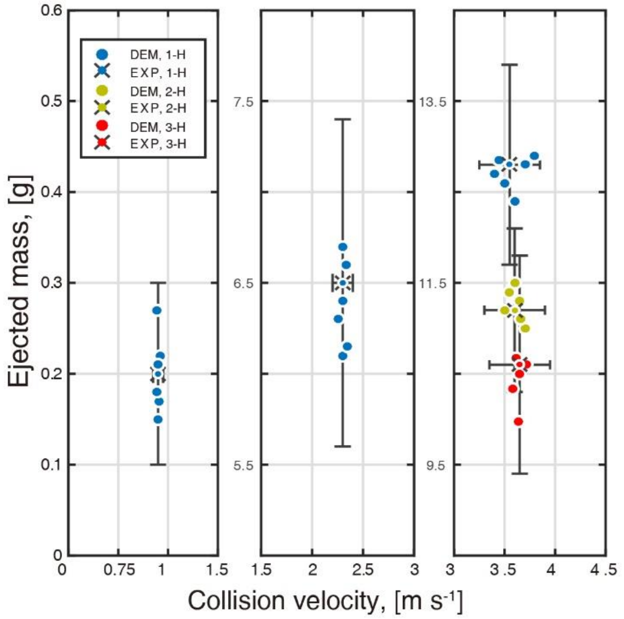

Supplementary Figure S2: Ejected mass for impacts into the granular bed with various height, i.e., 46.1 mm (blue), 92.2 mm (yellow) and 138.3 mm (red), at different velocities. The numerical results (dots) show a remarkable congruence with the experimental data (cross) within the measurement range.

Scaling law

The impact crater is the most commonly observed geological feature on the surface of solid Solar System bodies. The complex shapes and structures of these craters retain information on the past and present surface environments, as well as on the interior state[4]. Therefore, impact experiments on dry sand targets have been used to simulate an impact onto a regolith layer on celestial bodies, which yields some scaling laws for the impact phenomena based on laboratory experiments[5-7]. To show the physical plausibility of our code, we then numerically reproduce this empirical relation. We conduct a total of 12 simulations using a projectile particle (radius a = 3, 4, or 5 mm) vertically impacting into granular material targets that consist of 266,658 particles at velocity of v = 10, 30, 50, or 70 m s^{-1}. The projectile's material properties are assumed to be those of aluminum, and the granular target are assumed to be similar to quartz as used by Wada et al[4]. We prepare the target by randomly dropping the target particles into a container with diameter of 20 cm and height of 7 cm, and then propel a projectile particle from the top to impact vertically into the granular target.

As illustrated in Fig. S3, due to the penetration of the projectile, target particles are forcefully displaced due to high-velocity intrusions, consequently generating forced excavation flows. During this process, the cavity opens and expands with radial growth as observed in laboratory experiments[7]. As the projectile decelerates to rest, the motion of particles around the bottom of the cavity almost stops at this stage, whereas particles at the cavity wall still keep moving and the cavity continues to grow laterally. Afterwards, the gravitational collapse of a cavity eventually terminates, forming a final crater similar to that formed in laboratory experiments.

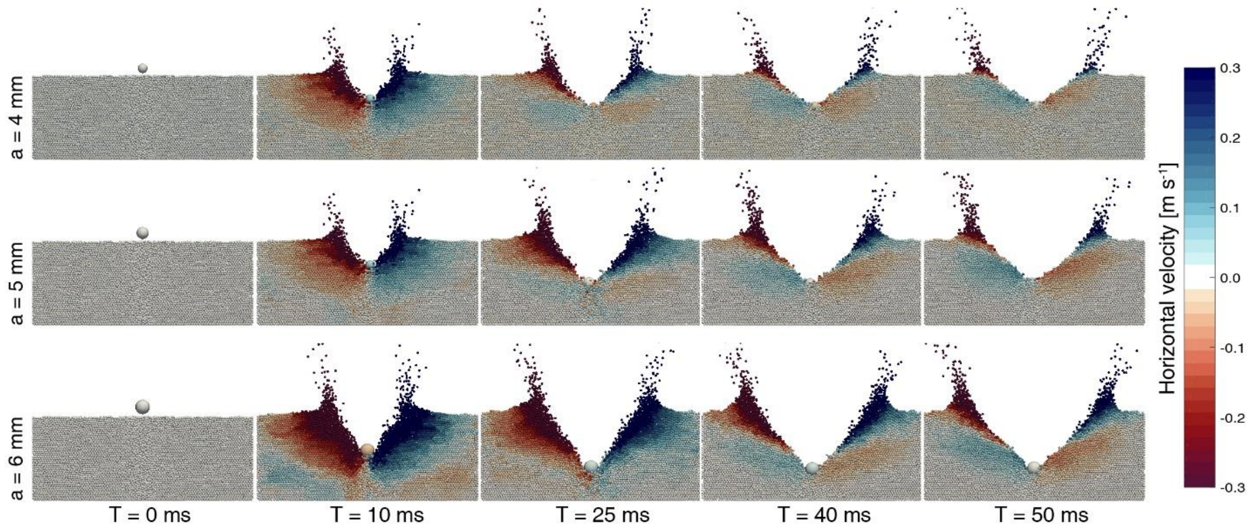

Supplementary Figure S3: Snapshots of the impacting process for various projectile radius, found by taking a vertical slice through the axisymmetric center of the simulated particle target as a function of time. The particles are colored depending on the horizontal velocity, as indicated in the scale bar.

In this test, the transient crater radius R is determined at the moment the bottom of the cavity begins to rise as suggested by Wada et al[4]. According to the π-group scaling law for the gravity regime[5], two dimensionless parameters are used: Π_R [$= R(\rho_t/m_p)^{1/3}$, the scaled-crater radius] and Π_2 [$= 3.22\, ga/v^2$, the gravity-scaled size], where ρ_t is the bulk density of the target and m_p, a, v are the mass, radius and velocity of the projectile, respectively. In Fig. S4, simulated data on the transient crater radius are plotted on a $\Pi_R - \Pi_2$ diagram, coupled with the scaling rules derived by the previous laboratory experiments[5-7]. The scaled-crater radii by our simulations are distributed on the line close to the scaling rules for quartz sand. Assume the power-law relation between Π_R and Π_2, we can determine the power-law exponents $\epsilon = -0.1742$ for the fitting data, where the value of correlation coefficient is 0.9988. The slope of our results is similar to that of the

experimental results for impact into quartz sand[5], -0.175; for impact into dry sand[6], -0.170; and for impact into soda lime glass grains[7], $-0.172 \sim -0.178$. Therefore, the excavation stage of cratering derived from experimental studies is represented well by our simulations. The impact simulation code developed in this study is thus suggested to be useful for the analysis of the impact cratering process on granular material.

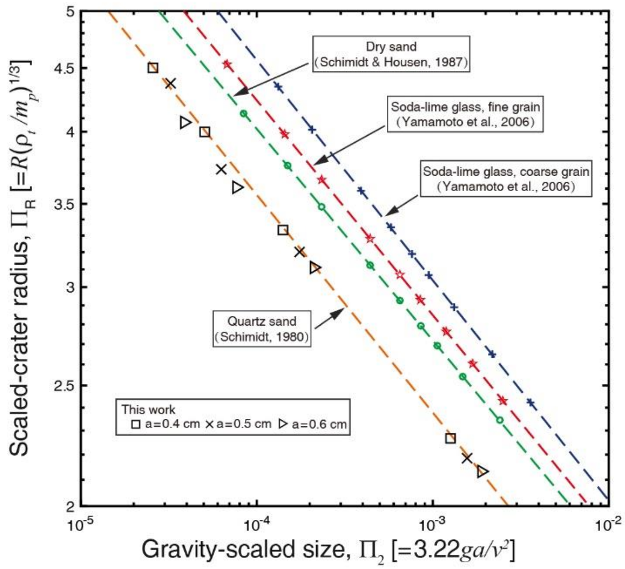

Supplementary Figure S4: The numerical results on $\Pi_R - \Pi_2$ diagram are plotted. For comparison, the π scaling lines obtained by laboratory experiments are shown by broken lines, including the impact data of quartz sand[5], dry sand[6] and soda lime glass grains[7].

Reference

1. Nedderman, R., Tüzün, U., Savage, S. & Houlsby, G. The flow of granular materials—I: Discharge rates from hoppers. *Chemical Engineering Science* **37**, 1597-1609 (1982).
2. Zhou, Y., Wright, B., Yang, R., Xu, B. & Yu, A. Rolling friction in the dynamic simulation of sandpile formation. *Physica A: Statistical Mechanics and its Applications* **269**, 536-553 (1999).
3. Li, Y., Dove, A., Curtis, J. S. & Colwell, J. E. 3D DEM simulations and experiments exploring low-velocity projectile impacts into a granular bed. *Powder Technology* **288**, 303-314, doi:10.1016/j.powtec.2015.11.022 (2016).
4. Wada, K., Senshu, H. & Matsui, T. Numerical simulation of impact cratering on granular material. *Icarus* **180**, 528-545, doi:10.1016/j.icarus.2005.10.002 (2006).
5. Schmidt, R. in *Lunar and Planetary Science Conference Proceedings.* 2099-2128.
6. Schmidt, R. M. & Housen, K. R. Some recent advances in the scaling of impact and explosion cratering. *International Journal of Impact Engineering* **5**, 543-560 (1987).
7. Yamamoto, S., Wada, K., Okabe, N. & Matsui, T. Transient crater growth in granular targets: An experimental study of low velocity impacts into glass sphere targets. *Icarus* **183**, 215-224 (2006).
